# Introduction of confidential enquiry into maternal deaths in Ethiopia: Implementation and methodological considerations

**DOI:** 10.3310/nihropenres.14170.1

**Published:** 2026-01-07

**Authors:** Sagni Girma, Thomas van den Akker, Redwan Ahmed, Mohammed Yuya, Jelle Stekelenburg, Tahir Ahmed Hassen, Delayehu Bekele, Marian Knight, Abera Kenay Tura

**Affiliations:** 1 College of Health and Medical Sciences, Haramaya University, Harar, Ethiopia; 2 Department of Obstetrics and Gynaecology, Leiden University Medical Center, Leiden, The Netherlands; 3 Vrije Universiteit Amsterdam Athena Instituut, Amsterdam, The Netherlands; 4 Department of Obstetrics and Gynecology, Hiwot Fana Specialized Comprehensive University Hospital, Harar, Ethiopia; 5 Department of Obstetrics and Gynecology, Medical Centre Leeuwarden, Leeuwarden, The Netherlands; 6 Department of Global Health, University Medical Centre Groningen, Groningen, The Netherlands; 7 School of Medicine and Public Health, The University of Newcastle, Newcastel, New South Wales, Australia; 8 Department of Obstetrics and Gynecology, St Paul's Hospital Millennium Medical College, Addis Ababa, Ethiopia; 9 National Perinatal Epidemiology Unit, Nuffield Department of Population Health, University of Oxford, Oxford, UK; 10 Department of International Public Health, Liverpool School of Tropical Medicine, Liverpool, UK

**Keywords:** Confidential enquiry, maternal deaths; maternal mortality; maternal death review; Ethiopia

## Abstract

**Background:**

Despite having high maternal mortality, no recent confidential enquiry into maternal deaths (CEMD) has been implemented in Ethiopia. This paper outlines the introduction of the CEMD, major findings, and key methodological considerations.

**Methods:**

We embedded this CEMD in the ongoing Ethiopian Obstetric Surveillance System (EthOSS), a regional system that monitors a range of major obstetric conditions in eastern Ethiopia. Multiple methods (both qualitative and quantitative) were used to collect, analyse and report the data. A multidisciplinary committee was established and trained on principles and methodology of CEMD by international experts. The CEMD committee conducted two plenary CEMD sessions to review maternal deaths reported from April 1, 2021, to March 31, 2022, from 13 hospitals in the EthOSS consortium. Each case was assessed for causes, contributing factors, delays in care using the three-delays model, preventability, and recommendations for improving care.

**Results:**

Out of 70 maternal deaths, in 59 there was enough information to enable a review by the committee; 27/59 (46%) and 15/59 (25%) were caused by obstetric haemorrhage and hypertensive disorders of pregnancy respectively. In 55/59 (93%), at least one of the three delays was identified: delay one (seeking care) in 48 (81%), delay two (reaching an appropriate facility) in 52 (88%), and delay three (receiving adequate care) in 54 (92%). The review indicated that almost all reported deaths could have been prevented with better care.

**Conclusions:**

Almost all the maternal deaths in the region were considered preventable. Training for improving providers’ clinical skills, improving availability of blood and basic supplies, strengthening postpartum monitoring, and referrals were recommended for saving lives through reducing preventable maternal deaths.

## Plain Language Summary

Maternal death is a tragedy that comes with pregnancy and childbirth complications. The consequences of death of a woman goes beyond her life and the family, and extends to the wider community and nation at large. We intended to identify common causes of maternal deaths at maternity units in eastern Ethiopia, and factors behind the medical cause of death through a confidential enquiry approach to learning from and prevent maternal deaths from similar causes.

The Ethiopian Obstetric Surveillance System (EthOSS) collected data on maternal deaths from 13 hospitals having maternity units and anonymised them before a review by the EthOSS committee for confidential enquiry in to maternal deaths (CEMD) on a quarterly meetings. The EthOSS study was approved by the Institutional Health Research Ethics Review Committee of the College of Health and Medical Sciences of Haramaya University in Ethiopia and the University of Oxford’s Oxford Tropical Research Ethics Committee. The reviews identified that majority of deaths were caused by direct obstetric causes; 27/59 (46%) by haemorrhage and 15/59 (25%) hypertensive disorders of pregnancy. Delay one (delay in seeking care), delay two (delay in reaching an appropriate health facility) and delay three (delay in receiving adequate care at health facility) were identified in 48 (81%), 52 (88%), and 54 (92%) cases reviewed. The reviews indicated that almost all maternal deaths were potentially preventable by appropriate care.

Based on the study findings, improving maternity care providers’ clinical skills through skill trainings, enhancing availability of blood and basic supplies, strengthening postpartum maternal monitoring, and strengthening inter- and intra-facility referrals were recommended for reducing preventable maternal deaths.

## Introduction

Despite substantial efforts and commitments, in 2023, an estimated 8, 000 women still died of complications during pregnancy, childbirth and the postpartum period in Ethiopia ^1^. Most maternal deaths resulted from common causes -hemorrhage, hypertensive disorders of pregnancy, sepsis, and anemia- all of which have well-established prevention and management strategies ^2^. The World Health Organization (WHO) introduced the Maternal Death Surveillance and Response (MDSR) program for countries with high number of maternal deaths in 2013 ^3^. Several low- and middle-income countries including Ethiopia adopted it ^4, 5^. Maternal death reviews, including those being part of MDSR, have been successful in countries such as the United Kingdom, South Africa and Malaysia ^6^. In other countries, including Kenya and Ethiopia, MDSR has come with challenges, including fear of being blamed among health workers and managers ^7– 9^. Efforts that ensure confidentiality of providers and health facilities are therefore essential facilitators for the success of death reviews ^9– 11^.

Maternal death reviews take different forms, from verbal autopsies and facility-based reviews, to complete national or sub-national confidential enquiry in to maternal deaths (CEMD). All types of review aim to identify causes of death and lessons to prevent similar deaths in the future ^6, 12^. CEMD is defined by the WHO as ‘a systematic multidisciplinary anonymous investigation of all or a representative sample of maternal deaths occurring at an area, regional (state) or national level, which identifies the numbers, causes and avoidable or remediable factors associated with them ^12^. Of the existing maternal death review types, CEMD has been recommended by the WHO as the most in-depth method. The CEMD can be part of the MDSR process in order to strengthen MDSR and arrive at more substantial messages for change, as indicated in the MDSR guidance ^3^.

Although Ethiopia has a history of CEMD being piloted in Addis Ababa in 1981–1983, CEMD has not been conducted since then ^13^. As part of the Ethiopian Obstetric Surveillance System (EthOSS) project, we introduced a regional CEMD system in eastern Ethiopia, aiming to learn lessons for a possible (re-)implementation at the national level ^14, 15^. In this paper, we describe the CEMD introduction process and report CEMD findings for deaths that occurred in all 13 hospitals in the EthOSS platform.

## Methods

### Patient and Public Involvement

Patients were not directly involved in the design of this study. Representative from ministry of health, heads of regional health bureaus, zonal health department, Ethiopian society of Obstetricians and Gynaecologists, Ethiopian Midwifery Association, Professional Association of Emergency Surgery Officers of Ethiopia, and people from academia were involved in the study as EthOSS Steering Committee. The steering committee reviewed the project progress, supervise and guide how the project activities should be done, approved the selection of EthOSS conditions to be investigated, and the design and data reporting forms of the project. Obstetricians, midwives, and critical care specialists were involved in the project launch and review meetings of EthOSS. The EthOSS methodology including EthOSS steering committee, case selection, reporting, data capturing and other details were previously published ^14^. Patients and the public were not involved in dissemination plan for the study.

### Study settings, design and period

This CEMD was introduced as part of the EthOSS project conducted by Haramaya University in collaboration with the University of Oxford in the United Kingdom, and Leiden University Medical Centre and University Medical Centre Groningen in The Netherlands, to adapt obstetric surveillance and confidential enquiry methodologies for use in a low-resource setting. Details pertaining to the adaptation of the methodology are described elsewhere ^14^.

In brief, EthOSS was introduced in 13 hospitals in eastern Ethiopia to report the number of women who had sustained any of five major obstetric conditions (obstetric hemorrhage, eclampsia, uterine rupture, sepsis, and severe anemia), as well as the number of women who died, using a system of voluntary reporting midwife based at each hospital. The level of hospital ranged from several lower-level primary (district) hospitals to a tertiary (comprehensive teaching) hospital ^14^. We used multiple methods (quantitative and qualitative) in collecting, analyzing and presenting data from this CEMD. Implementation of CEMD included the process of identification and anonymizing medical records of women who died, and establishing and training the CEMD committee, which subsequently reviewed all maternal deaths that occurred between 01 April 2021 to 31 March 2022.

### Maternal death identification

All maternal deaths in participating hospitals during the study period were identified and reported (n=70) on a monthly basis by a designated midwife. We obtained permission from respective hospitals to actively search records of deaths that were filed in the medical directors’ office. In many Ethiopian hospitals, this is the location where medical records of maternal deaths are temporarily retained to ensure that women’s deaths are documented for the purposes of MDSR, and ensure that no records are changed after a death occurred. In addition, registers of the central intensive care unit and all other adult wards where deaths might have occurred were searched. The full medical records of each woman were subsequently scanned and stored on a password-protected electronic file only accessible by the project team lead and the primary investigator.

### De-identification of maternal deaths

Upon receiving reports of maternal deaths in EthOSS, files for these women were scanned and deidentified centrally by the EthOSS team and stored for review by the CEMD committee. All personal identifiers, facility information, details of managing staff, and all other potentially identifiable details were removed before review by the CEMD committee. As an additional measure to ensure anonymity and confidentiality, individual CEMD committee members were not assigned to review maternal deaths that occurred in their own facilities except when they were being asked to clarify information.

### Training of confidential enquiry into maternal death committee

The CEMD committee was trained by experts from Mothers and Babies: Reducing Risk through Audits and Confidential Enquiries across the UK (MBRRACE-UK), the Netherlands Audit Committee for Maternal Mortality and Morbidity, and the College of Health and Medical Sciences, Haramaya University. A one-day hands-on training on principles and the process of implementing CEMD was provided for the 24 committee members. Members included obstetrician-gynecologists, midwives, one emergency and critical care physician, a critical care nurse, one anesthesiologist, emergency surgical and obstetric officers (cadres of associate clinicians), anesthetists, and public health experts. All CEMD committee signed informed consents to maintain confidentiality of patient information.

To ensure in-depth understanding of the entire CEMD process, the committee was provided with brief orientation on how to work with the CEMD template and practiced reviewing maternal deaths and near misses under the direct supervision of experienced trainers, followed by reflections on their experiences, including comparisons to their experiences with the routine MDSR process, which many members had previously been involved in. Recommendations on how to improve the overall CEMD process were actively sought from all committee members.

### The Ethiopian obstetric surveillance system confidential enquiry meetings

After maternal deaths case files were anonymized, a senior consultant obstetrician-gynecologist who was not actively in practice, but served in a managerial position and who fully supported the process, prepared case summaries for all maternal deaths. In addition to the case summaries, anonymized case files were shared with the committee members. Members of the CEMD committee then reviewed all deaths independently and recorded findings on the template prepared for this purpose, which was adapted from the MBRRACE-UK CEMD template.

Two CEMD plenary sessions were held for two days each, three months apart. Sessions were led by an independent chair and secretary. After individually reviewing the cases, each maternal death was reviewed in a plenary session to identify where improvements could be made. The plenary review of each death took an average of two hours. The CEMD committee members identified delays in care in the chain of events according to the three delays model by Thaddeus and Maine: delay one (delay in seeking care), delay two (delay in reaching an appropriate facility), and delay three (delay in receiving adequate care in the facility) ^16^.

### Statistical analysis

For quantitative outcomes, we used descriptive statistics, reporting means with standard deviations for continuous variables, and frequency and percentages for categorical variables. We calculated the maternal mortality ratio (MMR) (maternal deaths per 100, 000 live births) with its corresponding 95% confidence interval. Narrative descriptions of selected case scenarios (case vignettes) are presented alongside detailed discussion and recommendations from the CEMD committee to enable improvements to be made.

### Ethical approval

The EthOSS protocol was reviewed and approved by the Institutional Health Research Ethics Review Committee (Ref No. IHRERC/024/2021) of the College of Health and Medical Sciences of Haramaya University, Ethiopia; and the University of Oxford’s Oxford Tropical Research Ethics Committee (OxTREC Reference 530-21). All methods of this study were performed in accordance with the Declaration of Helsinki. Before the actual data collection, informed consent was obtained from the medical director of each hospital. All collected data were stored anonymously in a password-protected electronic file only accessible by the project team lead and the primary investigator.

### Consent statement

The CEMD committee members and data collectors were trained on major ethical issues, signed, informed consents of confidentiality and disclosed any conflicts of interest prior to participating in the review process. But, obtaining informed consent from individual woman as study participant was not applicable since this is review of case files or charts of women who died.

### Role of funding source

This research was funded by the Medical Research Council (MRC) to Marian Knight as part of the 2019 Global Maternal and Neonatal Health Funding Call [grant number: MR/T037962/1]. The funders had no role in study design, data collection, data analysis, data interpretation, or writing of the manuscript.

## Results

### Characteristics of study participants

During the study period, 34, 090 livebirths and 70 maternal deaths were reported to EthOSS from the 13 participating hospitals. The corresponding maternal mortality ratio was 205 (95% confidence interval 160–259) per 100 000 livebirths. A total of 59 maternal deaths were reviewed by the CEMD committee and included in this study. Deaths were excluded from review (n=11) if sufficient information is not available in the case files to enable the committee to perform a meaningful assessment, notably most commonly for deaths reported as *death on arrival*. Of the 59 maternal deaths reviewed, 42 (71%) were rural residents, 48 (81%) came to health facility by referral, and 39 (66%) women had no antenatal care booking. Only 13 (22%) of women who died were admitted to the intensive care unit and most deaths 47(80%) occurred during postpartum period ( Table 1).

**Table 1.** Characteristics of deceased women whose care was reviewed in eastern Ethiopia (n=59).

Variables	Category	n	%
Age (in years)	<20	3	5
	20–29	25	42
	30–40	31	53
Residence	Urban	17	29
	Rural	42	71
Referral status	Referred	48	81
	Not referred	11	19
Date of admission to health facility	Weekdays	47	80
	Weekend/holiday	12	20
Antenatal care booking	Yes	20	34
	No	39	66
Parity	1	16	27
	2–4	29	49
	≥ 5	14	24
Admission to intensive care unit	Yes	13	22
	No	46	78
Date of death	Weekdays	41	69
	Weekend/holiday	18	31
Death period	Antepartum	6	10
	Intrapartum	6	10
	Postpartum	47	80

### Causes of maternal deaths

**Figure 1.**
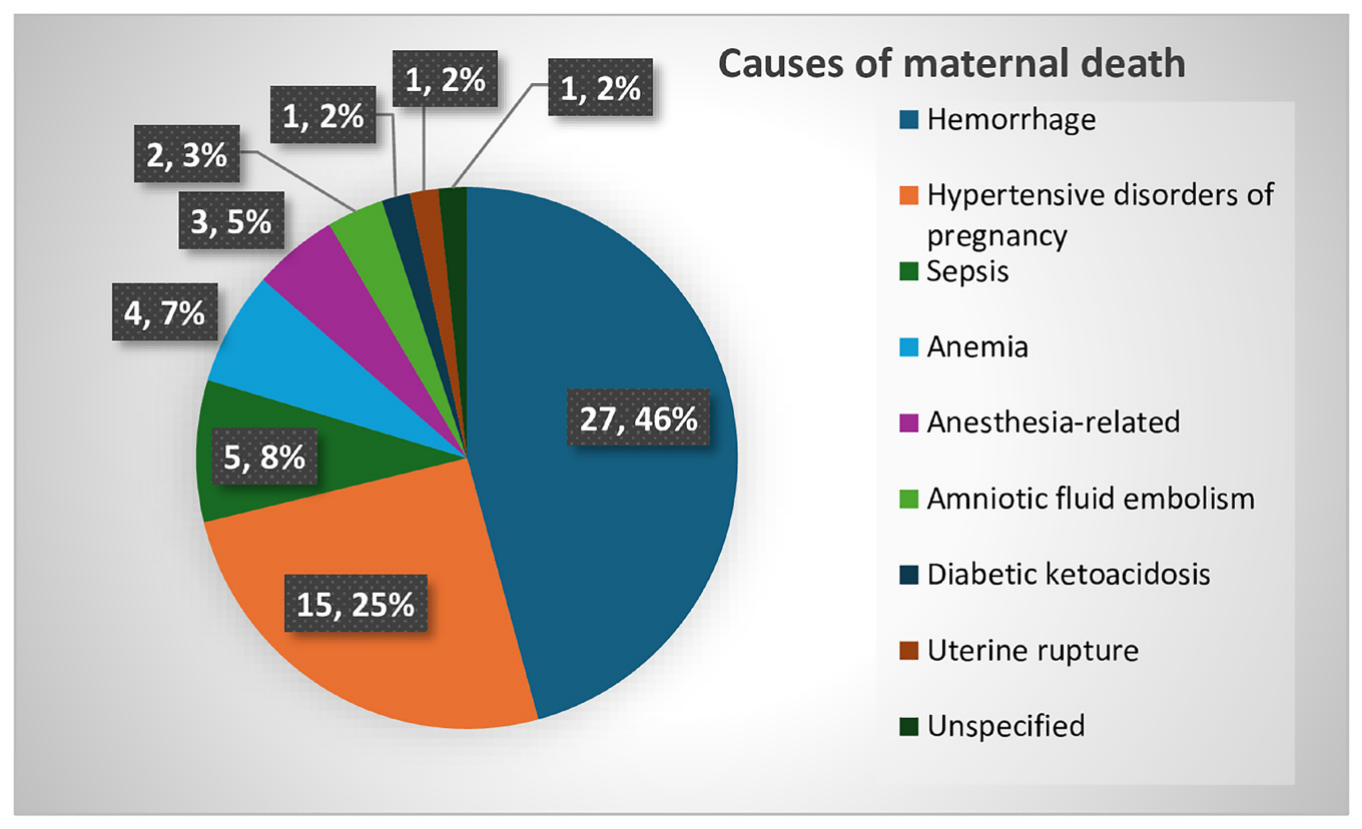
Causes of maternal deaths in eastern Ethiopia (n=59).

As shown in details in the Figure 1 below, almost all women’s deaths were categorized as direct: 27 (46%) from hemorrhage and 15 (25%) from hypertensive disorders of pregnancy, most commonly eclampsia.

### Factors contributing for maternal deaths

**Figure 2.**
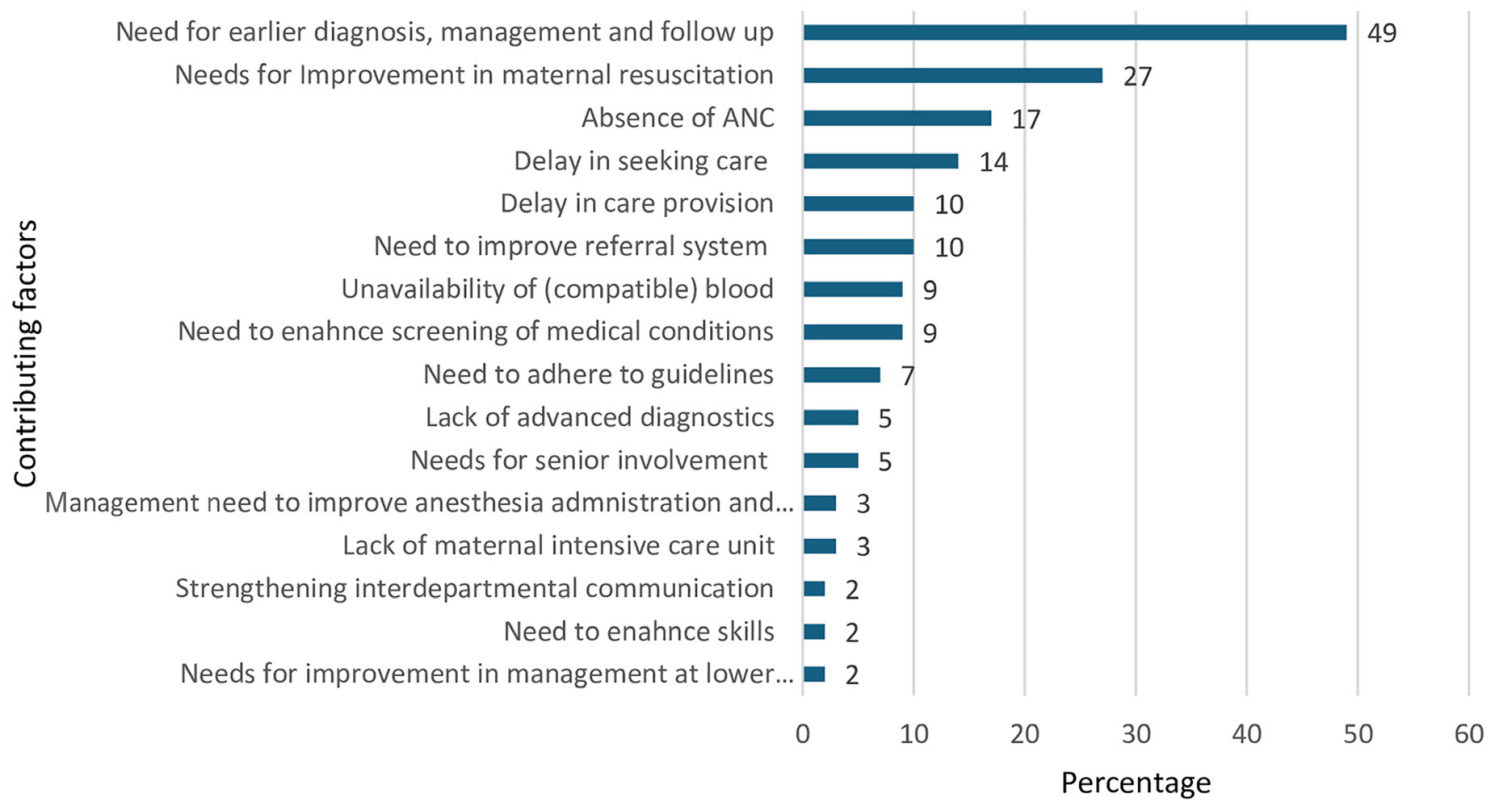
Distribution of factors contributing to maternal mortality in eastern Ethiopia (n=59).

As indicated in detail in Figure 2 below, the CEMD committee identified a total of 102 individual factors, grouped into 16 categories, that contributed to the 59 maternal deaths we included in this report. These factors were mainly related to diagnosis, management or follow up at the facility level 29/59 (49%), maternal resuscitation and stabilization 16/59 (27%), and absence of antenatal care 10/59 (17%).

Using the three-delay model, delay one, delay two and delay three were identified in 48/59 (81%), 52/59 (88%), and 54/59 (92%) of the deaths, respectively. Overall, in 55/59 (93%) of the deaths, one of the three delays was observed. In 58/59 (98%) of the deaths, improvements in care that may have made a difference in maternal outcome were identified.

### Lessons learned from selected case’s vignettes

***Referral problems***

In several instances there were lessons related to referral and woman transfer between health facilities (an example shown in *Vignette 1 below*).

**Vignette 1:** *A woman who gave birth to a stillborn baby in a nearby health center (a 10-minute drive) developed profuse vaginal bleeding postpartum. The team at the health center continued trying to manage the bleeding for 2 hours before referring her to the referral (tertiary level) hospital. Although she was in shock and unconscious upon arrival, she improved after abdominal packing, application of a non-pneumatic anti-shock garment, and receiving 4 units of blood, after which a hysterectomy was performed. She died 4 hours after the procedure in the Central Intensive Care Unit (CICU).*

In this example, the CEMD committee considered that the care she received at the higher level hospital was according to locally expected standards. It was agreed that earlier transfer to the hospital could have made a difference to her outcome. The referring health center is located within a 10-minute drive and there was no shortage of ambulances. The committee also pointed out that since this woman had an intra-uterine fetal death (IUFD), according to national protocol, she should have been referred to give birth at the hospital rather than being managed at the health center. Similar difficulties related to referral or transfer between facilities were noted in the care of four other women.

***Blood transfusion***

Lack of adequate (compatible) blood for transfusion is one of the main contributing factors for maternal deaths in 9% of women who died, as illustrated in Figure 2 and vignette 2 below.

**Vignette 2:** *A grand multiparous woman with a term pregnancy, who had not attended any antenatal care, presented to a tertiary hospital following vaginal bleeding for 13 hours. After giving vaginal birth to a stillborn baby, the bleeding continued. Uterotonics were given, a non-pneumatic anti-shock garment was applied, and only one unit of blood (the only available compatible blood) was transfused despite the severe blood loss, due to a lack of blood for transfusion. She went into shock and coma. No blood transfusion was available from any of the four blood banks in eastern Ethiopia and she died from severe bleeding.*

Although her blood group (O-negative) may have compounded the challenge to obtain blood for transfusion, this situation emphasizes the need for availing blood for transfusion in all centers providing comprehensive emergency obstetric care. Since she gave birth in the tertiary hospital, lifesaving interventions such as aortic compression, bi-manual uterine compression and of uterine balloon catheter should have been applied to stop further bleeding and avoid a higher demand for blood transfusion. There was no documentation of such efforts in the medical records. The team involved in the care of this woman also added the point that difficulty in finding blood donors in acute settings and a lack of blood screening facility at the blood bank nearby are commonly observed major challenges.

***Anesthesia-related***

In 3 (5%) women, complications related to anesthesia were identified as cause for maternal deaths reviewed in Figure 1 above (an example in Vignette 3) below.

**Vignette 3:** *A woman with a term pregnancy, who had antenatal care once during the pregnancy, presented to a primary (lower district level) hospital in labor. After pushing for 8 hours, vacuum extraction was tried but failed following which an emergency caesarean section was planned under spinal anesthesia. In the operating room, the anesthetist performed a lumbar puncture with a spinal needle, giving a regional spinal anesthetic upon the fourth attempt to reach the sub-dural space. Within five minutes of injection, the emergency surgical officer preparing for surgery noticed that the woman had a cardiorespiratory arrest. Resuscitation was tried using ambu-bag, intubation failed, and the woman died.*

This case represents a typical challenge of the existing gaps in administering a spinal anesthesia in lower-level facilities in absence of a fully functional mechanical ventilator. The CEMD committee involved the health care providers who participated in the management of this case, and identified a need for improvement in technical skills around intubation and resuscitation following spinal anesthesia. To address this gap, the EthOSS project team organized two rounds of training for intensive care unit nurses and anesthetists working in the hospitals under the EthOSS consortium followed by a week of practical learning at the emergency and critical care unit of the tertiary hospital, which is better equipped in terms of staffing. The training included safe administration of anesthesia, and basic life support around anesthesia.

***Indirect maternal deaths***

It was also identified that undetected underlying medical conditions of a woman during pregnancy are contributing factors to maternal deaths as highlighted in vignette 4 below.

**Vignette 4:** *A woman with a term pregnancy (primigravida) was referred from a primary hospital to a general (mid-level) hospital with complaints of shortness of breath. She was then diagnosed with severe anemia and congestive heart failure, and an intrauterine fetal death was found. She was subsequently referred to a tertiary hospital 200 km away for better medical management in Intensive Care Unit (ICU) care, but died during the ambulance transfer.*

This case represents an example of situation where priority should have been given to treating the medical needs of the woman rather than her obstetric condition. Although the general hospital is staffed with internal medicine specialist doctors, these had not been consulted. It was not planned to either provide pain medication or shorten the second stage of labor (or doing a destructive birth procedure). The diagnosis of congestive heart failure was made only very late in pregnancy at the general hospital. In addition, there appeared to be no clear need to refer her without stabilizing to a distant hospital with an intrauterine fetal death. The lack of a (fully functional) intensive care unit in the primary and general hospital might also have affected her management.

### Recommendations of the CEMD Committee to improve care

Based on the reviews, the CEMD committee identified a range of recommendations listed below for improvement in obstetric care and to prevent future maternal deaths.

1. All management actions should be well documented in the medical records.
2. Strengthen antenatal care screening and assess women with pregnancy-related risk factors in detail, and refer them antenatally or during early labor to higher level facilities.
3. Ensure that all maternity care providers, including those in lower-level health facilities, are equipped with basic life support skills such as cardiopulmonary resuscitation, by providing low-dose high-frequency in-service refresher training.
4. Ensure the availability of blood for transfusion in hospitals or nearby blood banks through enhanced blood collection from donors *.*
5. Ensure that general anesthesia is available for failed spinal anesthesia, as well as prompt resuscitation and mechanical ventilation in case of anesthesia-related complications *.*
6. Ensure that women with an intra-uterine fetal death are monitored, given proper medications (antibiotics), and screened for retained products of conception or bleeding after birth *.*
7. Stay alert that relatively minor hemorrhage can be fatal in presence of anemia. Ensure that a woman’s hemoglobin status is known and optimized during pregnancy *.*
8. Ensure that residents and/or midwives consult seniors promptly and that seniors are always available within a short time frame *.*
9. Strengthen interdepartmental communication and follow-up for pregnant and postnatal women around internal referral *.*
10. Ensure that mechanical ventilation and anesthesia staff are available where major surgery, including caesarean section, is practiced.

## Discussion

In this study, we report the process of establishing a regional CEMD and present findings from a review of maternal deaths that occurred from April 2021 to March 2022 in 13 hospitals in eastern Ethiopia. Overall, we included a review of 59 maternal deaths from two CEMD meetings held three months apart. Our study indicates that a majority of maternal deaths still result from direct obstetric causes although emerging indirect causes were also identified—an indication of the ongoing obstetric transition ^17^. After the confidential enquiry into maternal deaths in the 1980s in Addis Ababa ^13^, our study is the first of its kind in Ethiopia to report in such detail on causes of maternal deaths and their preventability.

In this CEMD, we found that the majority of maternal deaths were due to direct obstetric causes. This is in line with the national MDSR reports, where obstetric hemorrhage, hypertensive disorders of pregnancy and sepsis continued to be the leading causes ^2, 17, 18^. Whilst the causes of maternal deaths were similar to those identified in these MDSR reports, the in-depth CEMD identified additional system-level actions which could prevent future maternal deaths. Members of the CEMD identified several contributing factors leading to maternal mortality: delay in diagnosis, management or follow up; a need to improve resuscitation skills; and delay in receiving care (no antenatal care or late arrival), or delay in receiving appropriate care. Similar factors were also reported as contributors to maternal deaths from Kenya ^19^.

Given that the existing MDSR system is suffering from underreporting and an ineffective response ^7, 8^, scale up of CEMD might be essential to achieve the global target of less than 70 maternal deaths per 100 000 livebirths by 2030. MDSR reviews address facility-based maternal deaths or deaths in the community through a facility-based multidisciplinary committee. This approach is ideal for identifying rapid recommendations and contextualized responses ^12^. CEMD can complement this approach by deriving less obvious lessons and recommendations for change by a more thorough evaluation process ^12^. Thus, as recommended in MDSR technical guidance, CEMD is an approach that can complement an ideal MDSR process and strengthen maternal mortality surveillance ^3^. We felt that the motivation among CEMD committee members and their strong desire for change form an important impetus for a national roll out of CEMD in Ethiopia.

The use of the EthOSS platform enabled us to build the trust of respective hospitals to report their maternal deaths since they were convinced about the purpose of this program and its importance for improvements in obstetric care. This study was not with out limitations. Drawing the CEMD committee members from the already overburdened health care system in eastern Ethiopia was, however, one of the limitations. Although we worked hard to de-identify the case files so that names of facilities, providers and details of women were not traceable, complete anonymity might not have been achieved since only a limited number of facilities were included. Since members of CEMD were all health professionals, they might have focused more on delays two and three, and it is possible that factors contributing to delay one were underreported. Therefore, it is important to involve communities and relatives through verbal autopsy to look deeper into delay one. Moreover, poor documentation hampered the CEMD-process and prevented some deaths from being studied.

## Conclusions

Almost all the women’s deaths were considered preventable. Training for improving providers’ clinical skills, improving availability of blood and basic supplies, strengthening postpartum maternal monitoring, and strengthening inter- and intra-facility referrals were recommended for saving lives through reducing preventable maternal deaths.

## Data Availability

All data used for this study are available within the article. The data underlying this article cannot be shared publicly due to confidentiality and potential identification of sensitive individual patient information. The ethical approval of this study enforces keeping confidentiality of sensitive data. We have also signed a consent with administrators of the included hospitals in which we agreed to keep the data confidential. Requests for access to anonymized data can be directed to the EthOSS project at: mail@ethossnetwork.org and may be approved based on the discretion of the project team and/or the EthOSS steering committee. For the purpose of open access use of the article, the authors have applied a Creative Commons Attribution (CC BY) licence. The findings from the data were presented as research abstract at 33 ^rd^ annual conference of Ethiopian Society of Obstetricians and Gynecologists with a title; ‘Why women die: findings from a regional confidential enquiry into maternal deaths in eastern Ethiopia’ (available at: https://esog-eth.org/annual-conferences/).

## Software availability statement

Descriptive analyses were performed using Microsoft Excel and can be replicated.
