# Supplementary material for: Introduction of confidential enquiry into maternal deaths in Ethiopia: Implementation and methodological considerations
Source: NIHR Open Res. 2026 Jan 7;6:1. [Version 1] doi: 10.3310/nihropenres.14170.1 (PMC12881846; doi:10.3310/nihropenres.14170.1)
Supplement: Peer review history

# Reviewer report: Reviewer response for version 1

**DOI:** 10.3310/nihropenres.15421.r39327

**Published:** 2026-02-06

**Authors:** Abera Mersha Mamo

**Affiliations:** 1 Mizan-Tepi University, Mizan-Teferi, Ethiopia; 2 Arba Minch University (Ringgold ID: 128157), Arba Minch, South Ethiopia Region, Ethiopia

The study is technically sound and well-presented. However, to enhance clarity and align the manuscript with recent evidence, maternal death should be formally defined, and the latest maternal mortality reports from Ethiopia (2024–2025) should be integrated into the introduction.

 While the participant consent statement is clear, further explanation regarding institutional consent is required. Additionally, although the methods section specifies a mixed-methods approach and both sets of results are detailed in the main body, the abstract’s result section currently fails to highlight any key findings. Please ensure the abstract reflects the primary outcomes of both the qualitative and quantitative data.

Is the work clearly and accurately presented and does it cite the current literature?

Yes

If applicable, is the statistical analysis and its interpretation appropriate?

Yes

Are all the source data underlying the results available to ensure full reproducibility?

Yes

Is the study design appropriate and is the work technically sound?

Yes

Are the conclusions drawn adequately supported by the results?

Yes

Are sufficient details of methods and analysis provided to allow replication by others?

Yes

Reviewer Expertise:

I am a maternity and neonatal nursing specialist; I have worked on and published several studies related to maternal health issues. So, this is my area of expertise.

I confirm that I have read this submission and believe that I have an appropriate level of expertise to confirm that it is of an acceptable scientific standard.

# Reviewer report: Reviewer response for version 1

**DOI:** 10.3310/nihropenres.15421.r39183

**Published:** 2026-02-04

**Authors:** Farid Midhet

**Affiliations:** 1 Bahria University, Karachi, Pakistan

This very important study identifies the major weaknesses in the healthcare delivery system that may lead to a maternal death. Only the certified maternal deaths are included in the study for a confidential inquiry into each death by an expert committee. I believe that this study has achieved its stated objectives, and that it has extremely important lessons for the healthcare system in Ethiopia. The following are some suggestions to improve the validity and usefulness of the results:

1. Please clarify that the MMR calculated was hospital-based and may not reflect the overall MMR in the country/region.
2. Please confirm that the hospitals included in this study were open and accessible to the general population. This would ensure the readers that the cases included in the study are representative of the population served by these hospitals.
3. The three vignettes are interesting and provide and provide an insight into the individual stories. If possible, a few additional vignettes may be included.
4. Figure 2 should exclude the first category (need for earlier diagnosis, management, and follow-up) because it is too general and applies to nearly all deaths in the study. Removing this category would make the figure more meaningful.
5. The discussion and conclusion sections could be more useful by including the relevant steps to improve the quality of EMONC care (which is the central topic of this paper, although it is not explicit). These steps may include training of healthcare providers (give the areas where it is needed) in the hospitals and strengthening of the referral system.

Is the work clearly and accurately presented and does it cite the current literature?

Yes

If applicable, is the statistical analysis and its interpretation appropriate?

Partly

Are all the source data underlying the results available to ensure full reproducibility?

Yes

Is the study design appropriate and is the work technically sound?

Yes

Are the conclusions drawn adequately supported by the results?

Yes

Are sufficient details of methods and analysis provided to allow replication by others?

Yes

Reviewer Expertise:

Maternal and Neonatal Mortality. Population and Family Planning. Health Systems Research.

I confirm that I have read this submission and believe that I have an appropriate level of expertise to confirm that it is of an acceptable scientific standard.
